# Targeting Tumor Markers with Antisense Peptides: An Example of Human Prostate Specific Antigen

**DOI:** 10.3390/ijms20092090

**Published:** 2019-04-28

**Authors:** Nikola Štambuk, Paško Konjevoda, Petra Turčić, Hrvoje Šošić, Gorana Aralica, Damir Babić, Sven Seiwerth, Željko Kaštelan, Renata Novak Kujundžić, Piotr Wardega, Jelena Barać Žutelija, Ana Gudelj Gračanin, Mario Gabričević

**Affiliations:** 1 Center for Nuclear Magnetic Resonance, Ruđer Bošković Institute, Bijenička cesta 54, HR-10000 Zagreb, Croatia; 2 Laboratory for Epigenomics, Division of Molecular Medicine, Ruđer Bošković Institute, Bijenička cesta 54, HR-10000 Zagreb, Croatia; pkonjev@irb.hr (P.K.); rnovak@irb.hr (R.N.K.); 3 Department of Pharmacology, Faculty of Pharmacy and Biochemistry, University of Zagreb, Domagojeva 2, HR-10000 Zagreb, Croatia; 4 Department of Urology, Clinical Hospital Centre Split, Šoltanska 1, HR-21000 Split, Croatia; hsosic@gmail.com; 5 University Hospital Dubrava, Avenija Gojka Šuška 6, HR-10000 Zagreb, Croatia; garalica@kbd.hr; 6 Department of Pathology, School of Medicine, University of Zagreb, Šalata 10, HR-10000 Zagreb, Croatia; babic.damir@gmail.com (D.B.); seiwerth@mef.hr (S.S.); 7 Department of Pathology, University Hospital Centre Zagreb, Kišpatićeva 12, HR-10000 Zagreb, Croatia; 8 Department of Urology, University Hospital Centre Zagreb, Kišpatićeva 12, HR-10000 Zagreb, Croatia; zeljko.kastelan@gmail.com; 9 NanoTemper Technologies sp. z o.o., Bobrzynskiego 14, 30-348 Krakow, Poland; piotr.wardega@nanotempertech.com; 10 Clincal Department of Pathology “Ljudevit Jurak”, Clinical Hospital Center “Sestre milosrdnice”, Vinogradska 29, 10000 Zagreb, Croatia; jelena.barac@kbcsm.hr; 11 Department of Internal Medicine, Division of Clinical Immunology, Allergology and Rheumatology, School of Medicine, University of Zagreb, Dubrava University Hospital, Avenija Gojka Šuška 6, HR-10000 Zagreb, Croatia; agudelj@kbd.hr; 12 Department of General and Inorganic Chemistry, Faculty of Pharmacy and Biochemistry, University of Zagreb, Ante Kovačića 1, HR-10000 Zagreb, Croatia; mariog@pharma.hr

**Keywords:** neoplasm, prostate specific antigen, biomarker, antisense peptide, immunohistochemistry, nanomedicine

## Abstract

The purpose of this paper was to outline the development of short peptide targeting of the human prostate specific antigen (hPSA), and to evaluate its effectiveness in staining PSA in human prostate cancer tissue. The targeting of the hPSA antigen by means of antisense peptide AVRDKVG was designed according to a three-step method involving: 1. The selection of the molecular target (hPSA epitope), 2. the modeling of an antisense peptide (paratope) based on the epitope sequence, and 3. the spectroscopic evaluation of sense–antisense peptide binding. We then modified standard hPSA immunohistochemical staining practice by using a biotinylated antisense peptide instead of the standard monoclonal antibody and compared the results of both procedures. Immunochemical testing on human tissue showed the applicability of the antisense peptide technology to human molecular targets. This methodology represents a new approach to deriving peptide ligands and potential lead compounds for the development of novel diagnostic substances, biopharmaceuticals and vaccines.

## 1. Introduction

Classic methods of cancer therapy combine surgery, chemotherapy, hormonal therapy and radiation therapy [1]. Their main goal is the selective destruction of cancer cells, with minimization of toxicity in non-target tissues [1]. Targeted therapies, based on small organic molecules or monoclonal antibodies, have significantly improved the effectiveness of classic cancer therapy, while also being less harmful to normal cells [2]. However, the development and production of such therapies is often associated with high costs, with equally high costs in the application of therapy per individual patient [1,3]. 

Only a small number of molecular markers was exploited for development of targeted therapies, and one of the suggested approaches intended to improve production has been the use of small peptides [4]. Theoretically, small peptides for targeting of tumor markers could be used to develop a new generation of diagnostic and therapeutic substances [4,5]. Synthetic peptides have many advantages in comparison to monoclonal antibodies, i.e., relatively simple and inexpensive synthesis, stability and easy storage and transportation [6]. They show an absence of immunoreactivity, thus minimizing allergic side-effects [7,8]. Their small size gives them favorable pharmacokinetic properties, with better penetration to cancer tissue [5]. Ethical considerations are also significant, because the production of synthetic peptides does not require the sacrifice of animals or the application of procedures painful to them, in contrast to the production of monoclonal antibodies [9]. Peptides developed in this manner could be used for the development of radiopharmaceuticals for the selective targeting of primary and metastatic tumor markers, particularly useful in the detection of early metastases and staging of the disease [4,5].

This methodology thus represents a promising new way of deriving peptide ligands and potential lead compounds for the development of novel diagnostic substances, biopharmaceuticals and vaccines [10,11,12,13,14,15,16,17,18,19,20,21]. A similar approach has been used in biomedicine for the efficient modeling of more than 40 peptide–receptor systems; however, to our knowledge, immunohistochemical studies involving selective antigen imaging with antisense peptides are not available [11,12,13,14,15,16,17,18,19,20,21]. Therefore, in this paper we investigate whether a computationally derived short antisense peptide, targeting a selected epitope of the human prostate specific antigen (hPSA) could be used in preference to the standard monoclonal antibody for staining the PSA biomarker in human prostate cancer tissue [10,11,12,13,14,15,16,17,18,19,20,21,22].

## 2. Results and Discussion

The antisense peptide AVRDKVG was derived using amino acid pairing theory [5,19,20,21]. Following the selection of the molecular target (hPSA epitope), the potential peptide ligand was designed using antisense peptide translation in the 3′ to 5′ direction, and its binding affinity, measured as a dissociation constant (*K*_d_) was evaluated [10,11,12,19,20,21]. The peptide ligand obtained, AVRDKVG, was then biotinylated and used for hPSA immunohistochemical staining instead of the standard monoclonal antibody. The results of both methods were compared and discussed. 

### 2.1. Step 1: Selection of Molecular Target (hPSA Epitope) 

Previously published studies involving epitope scanning, phage libraries and antibody binding have shown that hPSA region, spanning from L50 to V65 (LLGRHSLFHPEDTGQV; Scheme S1), represents a well-defined epitope recognized by anti-PSA monoclonal antibodies (mAbs) [23,24,25,26]. The results of four classic studies, presented in Table 1, show that the septapeptide RHSLFHP (red), located in the region R53-P59 of hPSA, is a consensus epitope sequence [23,24,25,26].

**Figure 1:**
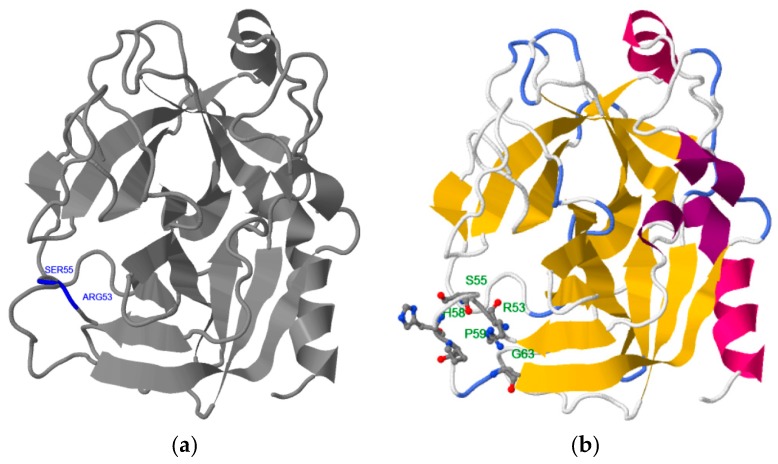
3D structure and predicted binding site of hPSA: (**a**) Phyre2 server, and (**b**) RaptorX server. hPSA sequence and protein data bank (PDB) information are presented in Scheme S1 and Scheme S2, respectively.

This finding is confirmed by different computational methods. Table 2 shows that the consensus of 10 epitope prediction servers, based on different physicochemical criteria [27,28,29,30,31,32,33,34,35,36,37,38,39,40], also points to the RHSLFHP sequence as the most probable hPSA epitope [23,24,25,26]. The same result is obtained using binding site prediction methods: Phyre2–3DLigandSite and RaptorX (Figure 1) [41,42,43,44]. The binding site predicted by Phyre2–3DLigandSite is in the vicinity of residues R53 and S55 (Figure 1a), while that predicted by RaptorX is close to R53, S55, H58 and P59 (Figure 1b). Therefore, hPSA epitope RHSLFHP was used as a starting point for the construction of the antisense peptide, paratope in Step 2.

### 2.2. Step 2: Modeling of Antisense Peptide AVRDKVG (hPSA Paratope)

**Scheme 1:**
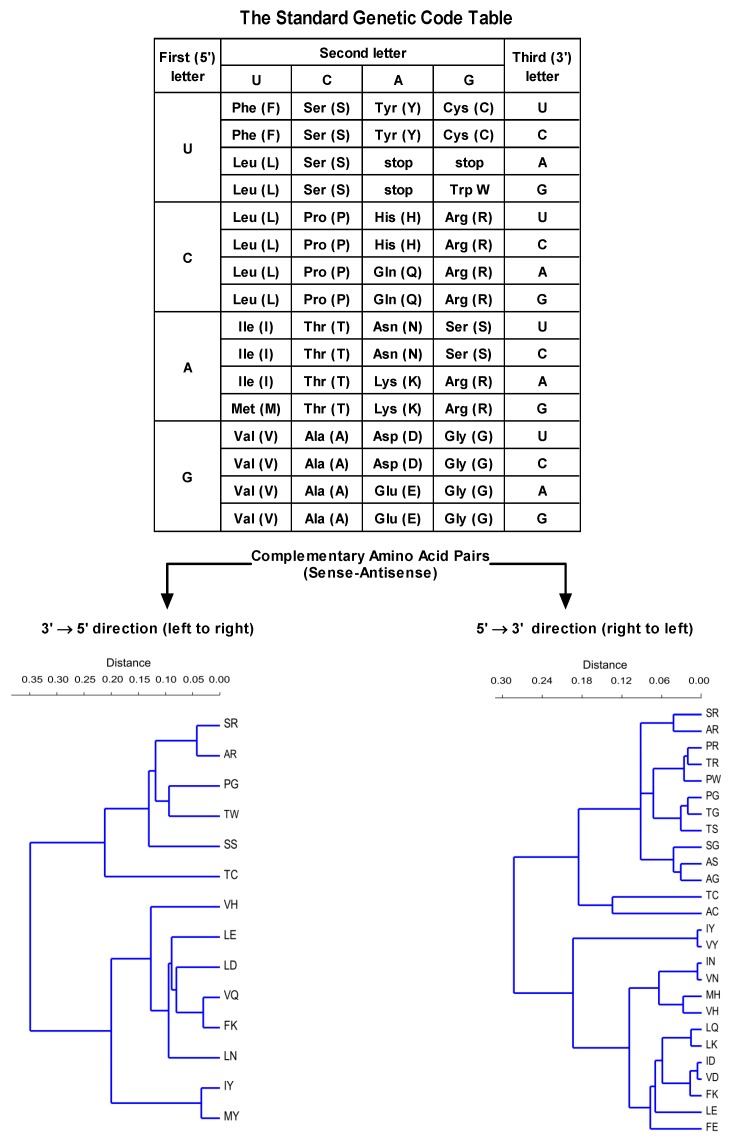
Twenty amino acids and three stop codons for the protein synthesis are specified by the genetic code, using sixty-four 3-letter codons (top). The number of antisense amino acids and related complementary pairs depends on the direction of codon translation (bottom) [19,20]. For the complementary (sense–antisense) pairs of the genetic code translated in a 3′ to 5′ direction *r* = 0.85, and for the complementary (sense–antisense) pairs of the genetic code translated in a 5′ to 3′ direction *r* = 0.86 (single linkage algorithm, Gower similarity index; PAST software 3.16).

The antisense peptide was designed using the translation of the hPSA epitope RHSLFHP in a 3′ to 5′ direction (Scheme 1) [19,20]. A 3′ to 5′ translation direction was employed since it produces fewer antisense peptides (Scheme 1) [19,20].

**Figure 2:**
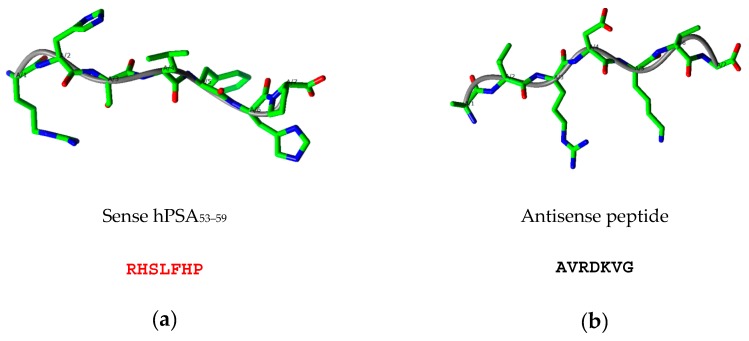
Predicted structures of (**a**) sense hPSA peptide epitope RHSLFHP (red) and (**b**) antisense peptide paratope AVRDKVG obtained with the PEPstrMOD method [46].

Table 3 illustrates the design of the antisense peptide AVRDKVG, obtained from the sense peptide epitope by the 3′ to 5′ translation of amino acid complementary pairs in Scheme 1 [19,20]. Potential antibody structures (paratopes) to hPSA epitope RHSLFHP were selected using the Basic Local Alignment Search Tool (BLAST), with the blastp, i.e., protein–protein, option [19,20,45]. The results presented in Table 3 show four antisense quadripeptide motifs detected in human antibody structures. The consensus (final) structure of the antisense peptide AVRDKVG was obtained as four linear paratope motifs AVRD, VRDK, RDKV and DKVG, selected on the basis of the highest score of antibody homologies detected by the BLAST search [19,20,45]. The predicted structures of the sense hPSA epitope RHSLFHP and its antisense paratope AVRDKVG using the PEPstrMOD method indicated extended structures that may facilitate binding (Figure 2, Schemes S3 and S4) [11,14,46].

**Figure A1:**
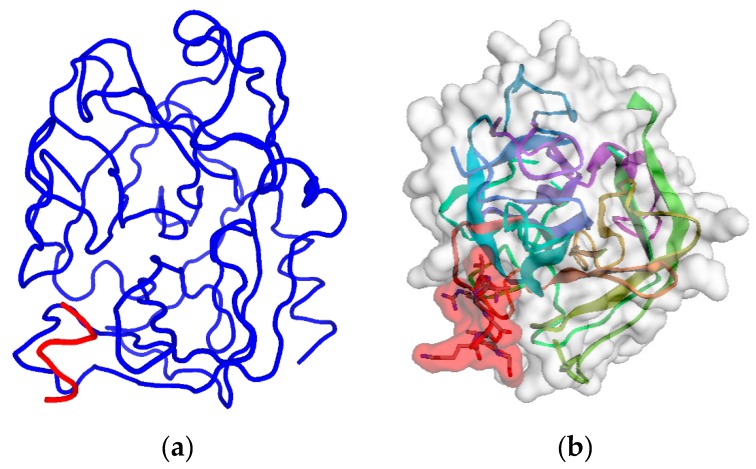
hPSA—AVRDKVG docking is accurately predicted using: (**a**) The pepATTRACT web server, and (**b**) CABS-dock server [47,48].

The protein-peptide interaction of hPSA region RHSLFHP and its antisense AVRDKVG was additionally investigated using pepATTRACT and CABS-dock web servers, which enable fully blind large scale in silico protein–peptide docking experiments [47,48]. The model No. 24 of pepATTRACT and the model No. 5 of CABS-dock, presented in Figure A1 and Table A1, confirm that the antisense peptide should bind to hPSA region RHSLFHP. Although based on different concepts [47,48], both methods yielded identical docking results, which indicates that the interaction of antisense paratope—AVRDKVG and hPSA epitope—RHSLFHP is highly probable. The coordinate file description obtained by the pepATTRACT and CABS-dock 3D models is given in Scheme S5 and Scheme S6, respectively.

### 2.3. Step 3: Spectroscopic Evaluation of Binding between Sense and Antisense Peptide

The affinity of epitope–paratope binding (*K*_d_) was experimentally verified in Step 3 with tryptophan fluorescence spectroscopy, microscale thermophoresis and magnetic particle enzyme immunoassay.

#### 2.3.1. Tryptophan Fluorescence Spectroscopy

**Figure 3:**
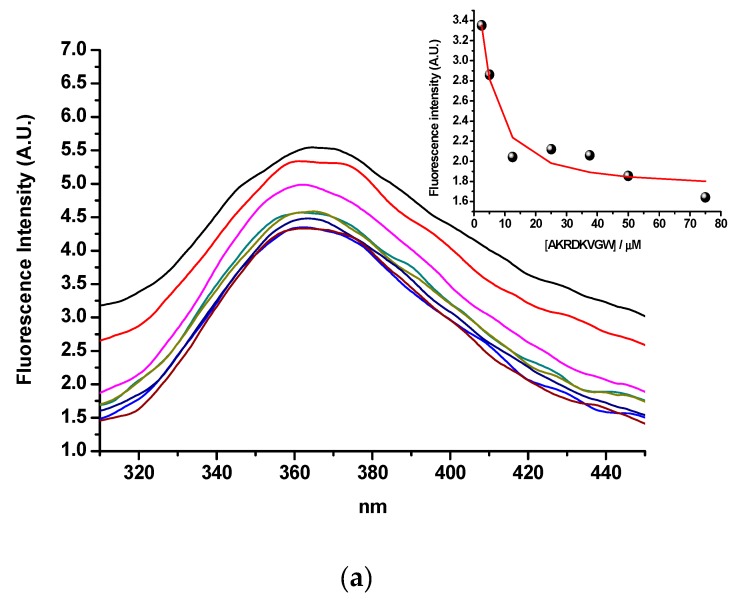

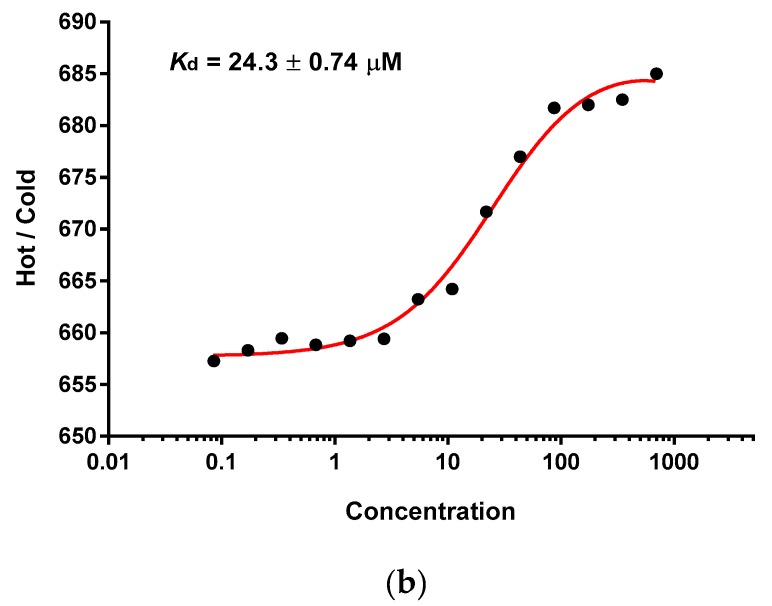
(**a**) Tryptophan fluorescence spectroscopy of the hPSA epitope RHSLFHP and its antisense ligand AVRDKVGW. Insert: Fitting curve at 360 nm. (**b**) Microscale thermophoresis of the hPSA epitope RHSLFHP and its antisense ligand Biotin-AVRDKVG.

The binding of hPSA peptide RHSLFHP and its antisense peptide AVRDKVG was evaluated by means of tryptophan fluorescence spectroscopy [19,20,49]. Tryptophan was added to the C-terminus of the antisense peptide (AVRDKVGW) to enable the fluorescence measurement. Singular value decomposition analysis suggested only two spectrally active species, one of the antisense paratope AVRDKVGW and the other of the complex of AVRDKVGW with its binding partner, RHSLFHP, since RHSLFHP was not spectrally active in fluorescence mode. The results in Figure 3a, suggest a 1-to-1 complex formation, without any higher order complexes. This model is given by equations (1) and (2), where *K*_d_ is the dissociation constant of the complex:

AVRDKVGW − RHSLFHP ⇄ AVRDKVGW + RHSLFHP (1)

(2) $$K_{d}=\frac{\left[\mathrm{AVRDKVGW}][\mathrm{RHSLFHP}\right]}{\left[\mathrm{AVRDKVGW}–\mathrm{RHSLFHP}\right]}$$

The calculated dissociation constant (*K*_d_) was 2.6 ± 0.19 µM (mean ± SEM), which indicates acceptable binding affinity for the epitope–paratope or antigen–antibody interaction [19,20,49].

#### 2.3.2. Microscale Thermophoresis and Magnetic Particle Enzyme Immunoassay

**Figure 4:**
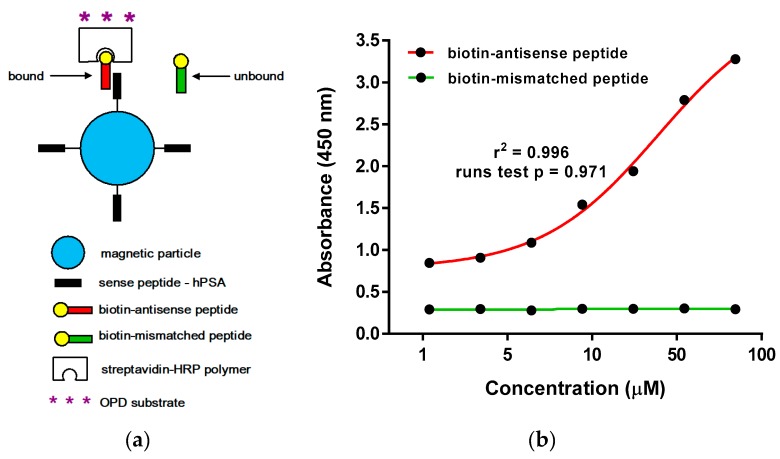
(**a**) Schematic representation of the magnetic particle enzyme immunoassay (MPEIA) based on the binding of sense hPSA peptide epitope coated onto SPHERO™ carboxyl magnetic particles and biotinylated antisense peptide paratope. (**b**) Detection of hPSA epitope RHSLFHP by magnetic particle enzyme immunoassay, using biotinylated antisense peptide AVRDKVG (red titration curve). Biotinylated mismatched peptide EHFRW was a negative control (green titration curve).

Biotinylation of the antisense peptide ligand AVRDKVG is a practical modification that enables the visualization of the ligand-acceptor complex with the secondary anti-biotin antibody (or streptavidin polymer) conjugated to HRP [20,50]. Consequently, microscale thermophoresis was used to analyze the binding of hPSA peptide RHSLFHP (residues 53–59) and its biotinylated antisense peptide AVRDKVG [19,20,50,51,52]. The data, presented in Figure 3b, showed a single binding event in a micromolar concentration range [19]. The dissociation constant of the complex was *K*_d_ = 24.3 ± 0.74 µM (Figure 3b; mean ± SEM). This indicated that N-terminal biotinylation of antisense peptide AVRDKVG, made for the purpose of immunohistochemical PSA staining, does not significantly alter the binding to hPSA region 53–59 (RHSLFHP). The results of the microscale thermophoresis were confirmed by the magnetic particle enzyme immunoassay (MPEIA), as shown in Figure 4.

We observed the binding of biotinylated antisense peptide AKRDKVG to sense hPSA peptide RHSLFHP coated onto SPHERO™ carboxyl magnetic particles in the range from 1.09 µM to 70 µM, which corresponds to the titration curve of the tryptophan fluorescence assay used for *K*_d_ determination in Figure 3a—Insert. The value *K*_d_ = 24.6 µM ± 0.20 µM measured by MPEIA (Figure 4b), was almost identical to the *K*_d_ values obtained by microscale thermophoresis (Figure 3b), i.e., both methods gave comparable results for the binding of hPSA epitope RHSLFHP and its antisense ligand Biotin-AVRDKVG. The insignificant drop in the binding affinity, resulting from biotin modification of the antisense molecule, was still within the micro- to millimolar *K*_d_ range adequate for the antibodies to exert their function [53,54,55]. hPSA epitope RHSLFHP that we used as a template for the antisense peptide binder (Biotin-AVRDKVG) was located in the epitope region II [23,24,25,26]. This well-known region of the PSA was exposed to the surface of the molecule, spanning from R53 to Q64 [23,24,25,26]. The specificity of the RHSLFHP binding was confirmed previously using anti-PSA mAbs and random peptide libraries [23,24,25,26]. For this research, a mismatched peptide Biotin-EHFRW was used as a negative control for the experimental design in Figure 4.

The measured dissociation constant of Biotin-AVRDKVG in the low µM range was appropriate for the most immunochemical applications, since weak affinity antigen–antibody recognition was shown to be efficient even at *K*_d_ > 10^−4^ M [53,54,55]. Strandh et al. have shown that, in addition to antibodies, surface plasmon resonance is also well suited for the low affinity *K*_d_ determination of small analytes with mw <1000 [54,55].

Our results in Figure 3b and Figure 4b confirm that microscale thermophoresis and MPEIA are also applicable for this type of assay, since the sense (RHSLFHP) and antisense (Biotin-AKRDKVG) peptides used in this study have the molecular masses of 893.02 and 970.15, respectively.

### 2.4. hPSA Immunohistochemistry with Biotynilated Antisense Peptide and Monoclonal Antibody

**Figure 5:**
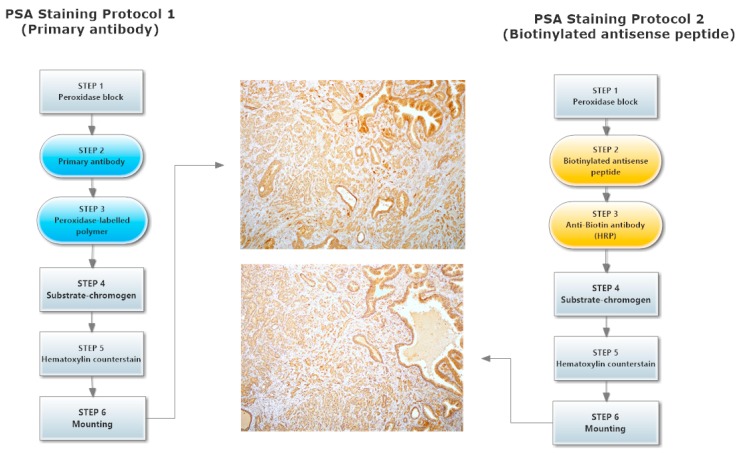
Two hPSA immunohistochemical staining protocols. The first is based on standard immunohistochemistry using a primary antibody (Protocol 1), while the second method is a novel procedure based on a biotinylated antisense peptide (Protocol 2).

**Figure 6:**
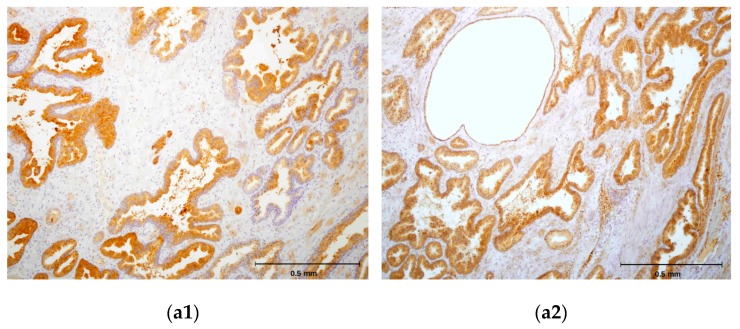

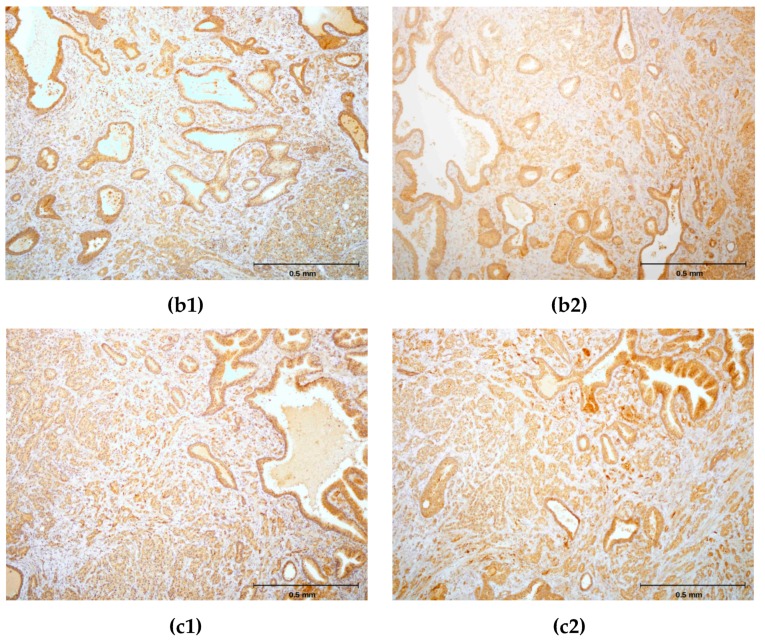
(**a1**,**b1**,**c1**) Immunohistochemical staining of three different hPSA tissue samples with standard monoclonal antibody ER-PR8 (dilution 1:100). (**a2**,**b2**,**c2**) Immunohistochemical staining of three different hPSA tissue samples with biotinylated antisense peptide AVRDKVG (dilution 1:100). The black line in the lower right corner denotes the scale bar (0.5 mm).

Direct testing of the antisense peptide binding to the hPSA molecule was carried out using human tissue samples, with two methods involved in the staining of hPSA (Protocol 1 and Protocol 2, Figure 5): The first staining protocol was based on standard immunohistochemistry using a primary antibody, while the second method was a novel procedure based on a biotinylated antisense peptide instead of the primary antibody (Figure 5 and Figure 6).

**Figure A2:**
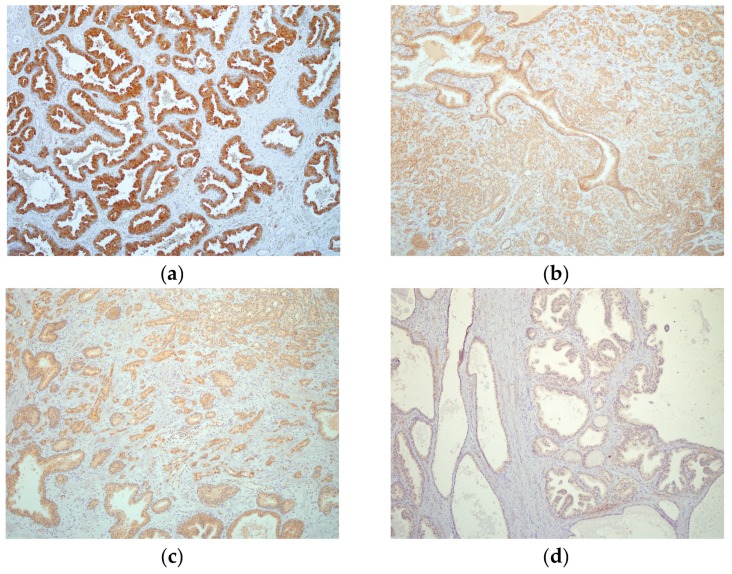
(**a**) Immunohistochemical staining of hPSA with standard monoclonal antibody ER-PR8 (1:100), compared to staining using antisense peptide Biotin-AVRDKVG in dilutions: (**b**) 1:50, (**c**) 1:100 and (**d**) 1:200.

**Figure A3:**
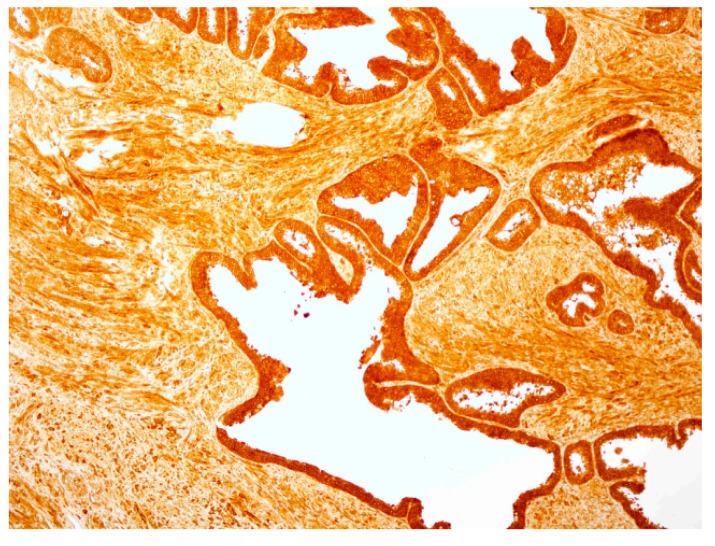
Antisense peptide Biotin-AVRDKVG applied in high concentration (1:10) produces increase in background, i.e., a non-specific staining of the prostate tissue.

Each protocol for PSA staining consisted of six steps. Steps 1, 4, 5 and 6 of protocol 1 and protocol 2 were identical, while steps 2 and 3 differed (Figure 5). In step 2 of protocol 2, a standard primary antibody was replaced by an N-terminal biotinylated antisense peptide AVRDKVG. In step 3 of protocol 2, the standard HRP-labelled polymer directed to the primary antibody was replaced by the HRP conjugated anti-biotin antibody. Otherwise the procedures were identical. The details of both protocols were presented in the Materials and Methods (Section 3.6). A comparison of these staining protocols conducted on parallel samples of seven different patients showed very similar staining of the hPSA antigen (Figure 6 and Table 4). Optimal antisense peptide dilution and standard monoclonal antibody dilution were very similar (Figure 6, Figure A2 and Figure A3).

It was confirmed that the antisense peptide AVRDKVG is comparable to the standard primary monoclonal mouse anti-human PSA antibody, with regard to the signal to noise ratio [56,57]. The best staining of the prostate cancer tissue was obtained with a dilution of 1:100, antisense peptide, which yielded a solid staining signal with minimal or absent background noise (Figure 6, Figure A2, Figure A3 and Table 4). Dilutions 1:10 and 1:50 of the antisense peptide showed an increase in background noise, while dilutions 1:200 and 1:500 were accompanied by a loss of staining signal (Table 4 and Figure A2). The specimens were analyzed using the current standards for immunohistochemical quality control (Table A2) [56,57], and the results are presented in Table 4. The overall quality of scoring according to the NordiQC criteria was good (score 2) [57].

**Figure 7:**
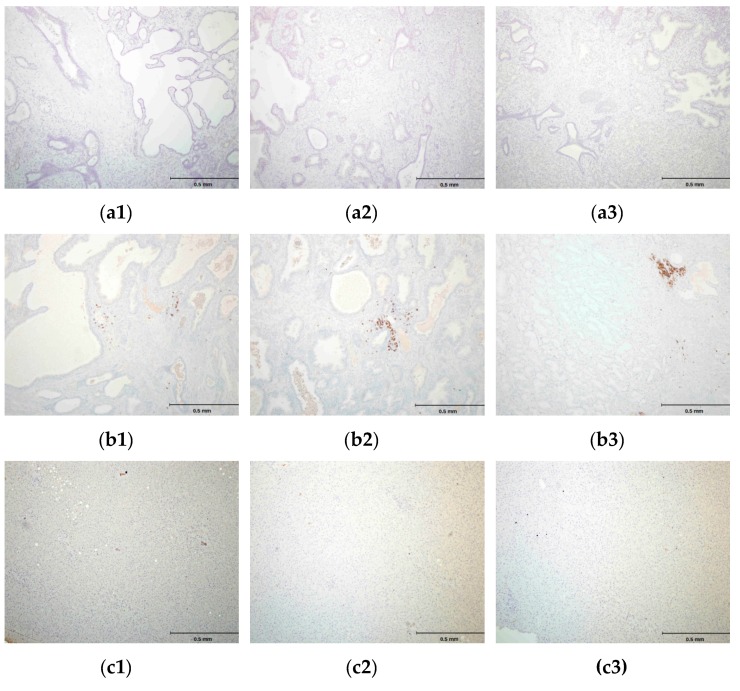
(**a1**, **a2**, **a3**) Negative control peptide: Immunohistochemistry (IHC) staining of the prostate tissue with a peptide Biotin-EHFRW that does not bind to hPSA epitope RHSLFHP. (**b1**, **b2**, **b3)** Negative control antibody―prostate tissue: IHC staining with CD20 (L26) monoclonal antibody; only inflammatory cells are stained. **(c1**, **c2**, **c3)** Negative control tissue―liver tissue: IHC staining with the antisense paratope Biotin-AVRDKVG that binds to hPSA epitope RHSLFHP. Scale bar = 0.5 mm.

It was not possible to perform the comparative analysis of PSA binding sites for Biotin-AVRDKVG antisense peptide and standard mouse-anti human PSA mAb ER-PR8 (Dako, Glostrup, Denmark), both used in this study for the immunohistochemistry (IHC) staining, since the location of ER-PR8 antibody epitope could not be established [58,59]. hPSA antisense Biotin-AKRDKVG, did not stain negative tissue control (liver), and its mismatched peptide Biotin-EHFRW and CD20 monoclonal antibody did not stain hPSA of the prostate tissue (Figure 7). Negative controls in Figure 7c1–c3 confirm the “technical specificity” of an IHC stain with Biotin-AKRDKVG, because there were no “false-positive reactions” in negative control tissue (liver tissue) [60]. For IHC performed on hPSA tissue section, “technical sensitivity” was demonstrated by evaluation of the intensity of the “stain reaction” (from weak to strong) in Table 4, Figure A2 and Figure A3 [60].

The results of this study suggest that antisense peptides could be used to target a specific human tumor biomarker, i.e., hPSA.

### 2.5. Concluding Remarks

Tryptophan fluorescence spectroscopy, microscale thermophoresis and magnetic particle enzyme immunoassay proved to be useful methods for determining the binding affinities of antisense peptides to their targets. Tryptophan fluorescence spectroscopy and microscale thermophoresis are simple, quick, inexpensive and could be used for high-throughput screening [19,20,49,51,52]. The combination of these methods with computational procedures for ligand-acceptor modeling and design enables the quick and simple selection of the antisense peptide structures, which can be used for further development [19,20,27].

**Figure 8:**
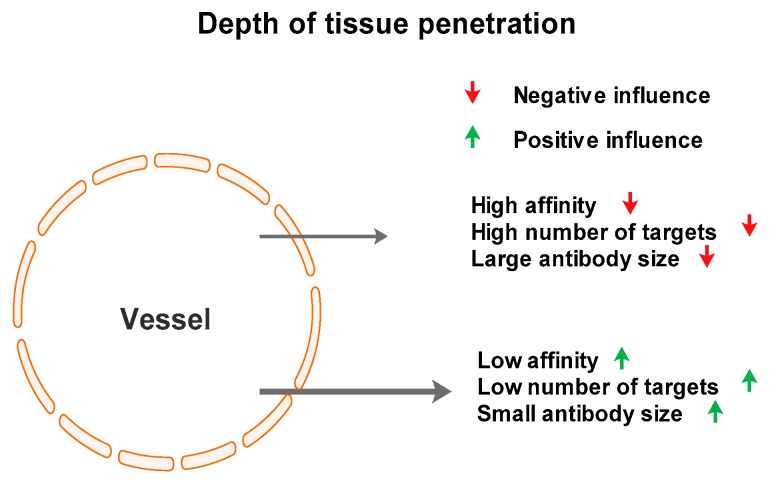
Factors influencing antibody penetration depth in the target tissue, according to Smith [61].

Our data suggest that selective targeting of tumor antigens could be achieved by using antisense peptides. This technology possesses certain advantages and disadvantages with respect to monoclonal antibodies. The most prominent difference is the small size of antisense peptides in comparison with antibodies. This property is very important for the depth of tissue penetration, especially in situations where peptide is intended to be used as a carrier, e.g., for radiopharmaceuticals or radiocontrast agents [5]. The *K*_d_ measurements for the hPSA antisense peptide used in our protocols is in the range of micromoles, whereas the value for monoclonal antibodies is in the nano- to micromolar range [61]. This suggests that maximum care must be applied to the selection of peptides with optimal affinity, i.e., the lowest possible *K*_d_ value. It is important to note that too high an affinity can, paradoxically, reduce the tissue penetration of antibodies [61]. In situations like this, very high-affinity antibodies will bind to targets near blood vessels (Figure 8), especially where the number of targets is high. This suggests that fluorescence spectroscopy, microscale thermophoresis and/or magnetic particle enzyme immunoassay should play a crucial role in the development of antisense peptide use, due to the fast, simple and inexpensive determination of *K*_d_ value.

Immunohistochemical staining is a logical step further in the use of antisense peptides, because it establishes selectivity in binding, especially in comparison with monoclonal antibody binding, which can be used as a gold standard. Immunochemical testing on human tissue directly shows the applicability of the antisense peptide technology to human molecular targets. The methodology of this preliminary study did not include pharmacokinetics, which is required for the development of diagnostic and therapeutic substances. Nevertheless, selected antisense peptides are potential lead compounds for the development of novel diagnostic, prognostic and therapeutic substances, biopharmaceuticals and vaccines [19,20].

## 3. Materials and Methods

### 3.1. Software Tools Used for Epitope and Ligand Binding Site Determination

In silico prediction of the linear hPSA epitope RHSLFHP was carried out using the consensus result of 10 servers presented in Table 2: ExPASy–ProtScale (Kyte & Doolittle hydropathy scale, window size 9), BcePred (antigenic propensity method), BepiPred-2 (random forest regression algorithm), LBtope (support vector machine models), SCRATCH–COBEpro (support vector machine based propensity score; Scheme S1), Sann and RVP-net (solvent accessibility algorithms), NetTurnP and LEPS (β-turn determination) and the informational spectrum method based on electron-ion interaction potential (ISM-EIIP) [27,28,29,30,31,32,33,34,35,36,37,38,39,40]. The hPSA sequence is given in Scheme S1.

The 3D structures of the hPSA molecule and its binding sites were predicted by coupled Phyre2–3DLigandSite servers for protein modeling, prediction and analysis (Figure 1a) [41,42]. 100% of hPSA residues were modeled at >90% confidence, the 3D structure template, obtained with X-ray diffraction, was protein data bank (PDB) file 2ZCH chain P (Scheme S2) [41]. The results obtained by Phyre2–3DLigandSite-based prediction were confirmed by RaptorX web portal Binding Prediction (Figure 1b) [44].

The antisense peptide, i.e., paratope AVRDKVG, was designed by the translation of the RHSLFHP epitope in a 3′ to 5′ direction (Scheme 1) [19,20]. Potential paratopes to hPSA_53–59_ epitope were selected using BLAST, with the blastp quadripeptide option (protein–protein BLAST) [19,20,45].

The 3D structures of the sense epitope RHSLFHP (Scheme S3) and its antisense paratope AVRDKVG Scheme S4), presented in Figure 2, were modeled using PEPstrMOD method [46]. The PDB files were visualized using CCP4 software version 7.0 [62].

Protein–peptide interactions between hPSA and its antisense peptide AVRDKVG were investigated using the pepATTRACT and CABS-dock web servers [47,48]. Both web services required the hPSA (protein) input file in PDB format (Scheme S2) and docking peptide sequence (AVRDKVG).

The pepATTRACT server is a novel fully blind docking protocol that does not require any information about the binding site [47]. In a very short time (~10 min) this web server returns an analysis of the most prevalent protein–peptide contacts among the top 50 generated models [47]. The pepATTRACT protocol is well tuned to assist users to identify the binding site, performs large-scale in silico experiments, and represents a useful starting point to rationalize the design for further protein–peptide docking experiments [47]. Model No. 24, presented in Figure A1a, is a precise description of hPSA–AVRDKVG docking. Results are given in PDB format as above (Scheme S5).

The CABS-dock server enables the modeling of protein–peptide interactions through an efficient method for the flexible docking of peptides to proteins without pre-defining the localization of the binding site [48]. The CABS-dock protocol was tested over the largest dataset of non-redundant protein–peptide interactions available to date, which includes bound and unbound docking cases [48]. Using a ligand RMSD cutoff of 5.5 Å, the best 10 models are selected with reference to the quality of docking models, which represent low-to-medium-accuracy models [48]. Model No. 5, presented in Figure A1b and Table A1, gives a precise description of hPSA–AVRDKVG docking. This result was given in the PDB format as Scheme S6.

### 3.2. Peptides Used in Experiments

1. Human prostate specific antigen (hPSA) peptide 53–59: RHSLFHP (mw 893.02, >97% purity, GenScript, Piscataway, NJ, USA).
2. C-terminal tryptophan antisense peptide: AVRDKVGW (mw 930.07, >97% purity, GenScript, Piscataway, NJ, USA).
3. N-terminal biotin antisense peptide: Biotin-AVRDKVG (mw 970.15, >97% purity, GenScript, Piscataway, NJ, USA).
4. N-terminal biotin peptide: Biotin-EHFRW (mw 1000.14, >97% purity, GenScript, Piscataway, NJ, USA).

### 3.3. Tryptophan Fluorescence (Binding of hPSA Epitope RHSLFHP and its Antisense Peptide)

The fluorescence spectra of the sense and antisense peptides, and their complexes, were measured at 25 °C by OLIS RSM 1000F spectrofluorimeter (Olis, Inc., Bogart, GA, USA) equipped with a thermostatted cell holder. The excitation wavelength was 280 nm (Figure 3a) [19,20,49]. The antisense peptide AVRDKVGW became bound to the hPSA peptide RHSLFHP, and their complex exhibited fluorescence, whereas the ligand RHSLFHP did not. The fluorescence units in Figure 3a are given as a ratio of signals obtained from sample and reference photomultiplier tubes (PMTs). SPECFIT software was used to analyze data obtained from the titrations [19,20,49]. The titration curve, in Figure 3a―Insert: Fitting curve at 360 nm, shows a good fit to data (*r*^2^ = 0.947). The dissociation constant was 2.6 ± 0.19 µM (*K*_d_ = mean ± SEM).

### 3.4. Microscale Thermophoresis (Binding of hPSA Epitope RHSLFHP to its Antisense Peptide)

The binding between the biotinylated antisense peptide AVRDKVG and a titrant, i.e., hPSA peptide RHSLFHP (Figure 3b), was observed by the method of microscale thermophoresis (MST) [19,20,51,52]. MST-analysis was performed using the Monolith.NT.115 instrument (NanoTemper Technologies GmbH, Munich, Germany). Biotin-AVRDKVG antisense directed to the hPSA region 53–59 was labeled with DY-495 (Red). The labeling procedure and the subsequent removal of unattached dye were performed within 45 min. A serial dilution of the non-labeled hPSA titrant was prepared in a 10 mM phosphate buffer. The concentration of biotin-hPSA antisense labelled with DY-495 was kept constant, while the concentration of the non-fluorescent binding partner (hPSA peptide RHSLFHP_53–59_) was varied between 0.0854 µM and 2.8 mM. After a short incubation, the samples were loaded into MST NT.115 standard glass capillaries and the MST-analysis was performed [19,20,51,52]. The data were analyzed using GraphPad Prism software version 5. The titration curve shows a good fit to data (*r*^2^ = 0.989), *K*_d_ = 24.3 ± 0.74 µM (mean ± SEM).

### 3.5. Magnetic Particle Enzyme Immunoassay (Binding of hPSA Epitope RHSLFHP and its Antisense Peptide)

SPHERO™ Carboxyl Magnetic Particles (2.5% *w*/*v*, 1.14 μm) were coated with hPSA peptide (RHSLFHP) using a slightly modified one step 1-ethyl-3-(3-dimethylaminopropyl) carbodiimide (EDC) coupling method suggested by manufacturer [20,50]. Briefly, the reaction mixture was composed of 2 mL sodium acetate buffer (0.01M, pH 5.0), 2 mg of peptide and 2 mL of 2.5% *w*/*v* carboxyl magnetic particles―1.14 μm, 20 mg of EDC. Reactions proceeded in glass reaction tubes overnight at room temperature in a rotary mixer. Tubes were centrifuged at 3000× *g* for 15 min, supernatant was carefully discarded, and the pellets were washed twice in 4 mL isotonic buffered saline (IBS), followed by centrifugation [20,50]. After washing, coated magnetic particles were resuspended in 4 mL of PBS containing 0.05% Tween 20.

Two folds serial dilutions (1.09–70 µM) of biotinylated antisense peptide AVRDKVG were made in 96-well microtiter plates to generate standard curves (Figure 4b). Mismatched peptide Biotin-EHFRW was used as a negative control (Figure 4b). A suspension (1.25% *w*/*v*) of carboxyl magnetic particles, coated with sense peptide RHSLFHP, was added to each well (40 µL/well) [20,50]. The plates were incubated at room temperature for 30 min and washed with PBS-Tween 20 five times. The washing solution was removed each time using the Spherotech UltraMag Separator (Sperotech, Inc., Lake Forest, IL, USA).

A blocking solution (PBS with 1% BSA) was added to the wells and incubated at room temperature for one hour. Wells were washed (PBS with 0.05% Tween 20) and ultrasensitive streptavidin-peroxidase polymer conjugate, diluted in the ratio 1:200 in PBS with 0.05% Tween 20, was added to the wells and incubated at room temperature for one hour (S 2438, SIGMA^®^, Saint Louis, MO, USA). Wells were washed five times using PBS with 0.05% Tween 20. SIGMA*FAST*™ OPD solution (o-phenylenediamine dihydrochloride) was added to the wells and incubated, in the dark, for 30 min at room temperature. The absorbances were read at 450 nm on a multiwell plate reader. The data were analyzed using GraphPad Prism software version 5. The titration curve in Figure 4b shows a good fit to data with *r*^2^ = 0.996, and *K*_d_ = 24.6 ± 0.20 µM (mean ± SEM).

### 3.6. Immunohistochemical Staining of hPSA: Protocol 1 and Protocol 2

The study was conducted in accordance with the Declaration of Helsinki, and the protocol was approved by the Ethics Committee of the University Hospital Centre Zagreb, No. 01-20/32-1-2006 (approved 28 February 2006). The project code of the Croatian Ministry of Science and Education was 098-0982929-2524.

Immunohistochemistry was performed on deparafinized slides (xylen and a series of ethanol solutions) at room temperature (20–25 °C). The phosphate-buffered saline (PBS) was used, with a pH of 7.2 and containing disodium hydrogen phosphate, sodium chloride, potassium chloride and potassium dihydrogen phosphate was used. PBS stock solution: 4 g KH_2_PO_4_, 23 g Na_2_HPO_4_, 4 g KCl, 160 g NaCl and 810 mL distilled water. Before staining, 100 mL of stock solution was diluted with 1900 mL of distilled water.

All slides (from seven patients) were selected from the repository of prostate cancer tissue, with diagnosis confirmed by consensus of two independent pathologists. The two pathologists who examined the slides stained with antisense peptides were unconnected with the pathologists who established the initial diagnosis.

Each of the two immunohistochemistry protocols for PSA staining consisted of six steps. Steps 1, 4, 5 and 6 of protocol 1 and protocol 2 were identical, while steps 2 and 3 differed (Figure 5 and Figure 6). For this study, the slides were kept in the dark at room temperature (20–25 °C), to prevent fading as a result of exposure to strong light. They were analyzed using an Olympus BX41 microscope, with an Olympus DP71 camera set to three levels of magnification: less than 40×, 100×, and 500×.

#### 3.6.1. Protocol 1 (Standard IHC Staining Technique)

Staining protocol 1 is a standard IHC staining technique using commercially available kit from Dako (Dako EnVision+System-HRP (AEC), Glostrup, Denmark) and primary monoclonal mouse anti-human PSA antibody. The kit and primary mouse antibody are intended for the quantitative identification of hPSA by light microscopy (Figure 5). For this study the endogenous peroxidase activity was first quenched by incubating the specimen with Dako’s Peroxidase block (step 1). Then, the specimen was incubated with diluted mouse primary antibody, followed by incubation with the HRP labeled polymer conjugated to goat anti-mouse immunoglobulins―steps 2 and 3, respectively. The staining was completed by incubation with AEC + substrate-chromogen (step 4), followed by hematoxylin counterstain and mounting―step 5 and 6, respectively. The negative control was the primary monoclonal mouse anti-human CD20 (Clone L26; Dako, Glostrup, Denmark).

IHC staining―protocol 1 was performed in accordance with the manufacturer’s instruction, as follows:

1. The specimen was covered with peroxidase block, i.e., 100 μL of 0.03% hydrogen peroxide containing sodium azide (Dako, Glostrup, Denmark), and incubated for four minutes. Following this procedure, the specimen was gently rinsed with phosphate buffered saline (PBS), and placed in a fresh buffer bath.
2. Primary monoclonal mouse anti-human PSA antibody (Clone ER-PR8, Code M0750; Dako, Glostrup, Denmark) was diluted with standard diluent containing 0.05 mol/L TRIS-HCl buffer and a 1% bovine serum albumin (BSA). 100 μL of primary antibody diluted in the proportion 1:100 was used to cover the specimen. After 30 min of incubation at room temperature, the specimen was gently rinsed with PBS and placed in a fresh buffer bath.
3. 100 μL of HRP-labelled polymer conjugated to goat anti-mouse immunoglobulins in Tris-HCl buffer containing stabilizing protein and an anti-microbial agent (Dako, Glostrup, Denmark) was applied to cover the specimen, followed by a 30 min incubation. After 30 min of incubation, the specimen was gently rinsed with PBS and placed in a fresh buffer bath.
4. The specimen was covered with 100 μL of AEC + substrate-chromogen solution for 10 min, i.e., 3-amino-9-ethylcarbazole containing hydrogen peroxide, stabilizers, enhancers and anti-microbial agent (Dako, Glostrup, Denmark). After that period the specimen was again rinsed with PBS.
5. The slides were immersed in a bath of aqueous hematoxylin (Mayerr’s hematoxylin), and rinsed gently in a distilled water bath. Slides were dipped 10 times into a bath of ammonia (0.037 mol/L), and rinsed in a bath of distilled water for four minutes.
6. The specimens were mounted and coverslipped with the non-aqueous permanent mounting medium Ultramount.

#### 3.6.2. Protocol 2 (Modified IHC Staining Using Antisense Peptide Instead of Primary Antibody)

The staining protocol 2 was a modified IHC protocol 1, as follows: In step 2 we used antisense peptide Biotin-AVRDKVG, instead of the primary monoclonal mouse anti-human PSA antibody, in step 3 we used rabbit polyclonal antibody to biotin conjugated to HRP, instead of HRP labeled polymer conjugated to goat anti-mouse immunoglobulins (Figure 5), with peptide Biotin-EHFRW as a negative control (Figure 7). Otherwise, the procedures and chemicals were identical. Quality control for the PSA staining was investigated using NordiQC scoring criteria [57]: 0—Poor; 1—Borderline; 2—Good; 3—Optimal. The scale is presented in Table A2, and the final results given in Table 4 are expressed as a sum of scores.

IHC staining―protocol 2:

1. The specimen was covered with peroxidase block, i.e., 100 μL of 0.03% hydrogen peroxide containing sodium azide (Dako, Glostrup, Denmark), and incubated for four minutes. Following this, the specimen was gently rinsed with phosphate buffered saline (PBS), and placed in a fresh buffer bath.
2. Biotinylated antisense peptide AVRDKVG (GenScript, Piscataway, NJ, USA) directed to PSA epitope 53–59 (RHSLFHP) was used to cover the specimen. Five milligrams of Biotin-AVRDKVG antisense was diluted in 2.5 mL PBS. Dilutions 1:10, 1:50, 1:100, 1:200 and 1:500 were applied to cover the specimen. A 100 μL volume was used per section. After 30 min of incubation, each specimen was gently rinsed with PBS and placed in a fresh buffer bath.
3. Anti-biotin antibody conjugated to HRP (ab34645, Abcam, Cambridge, UK) was applied to cover the specimen. Of the antibody 10 mg/2 mL was diluted 1:100. One hundred microliters of the diluted antibody was applied to cover each specimen, followed by a 30 min incubation. After the incubation, the specimen was gently rinsed with PBS and placed in a fresh buffer bath.
4. The specimen was covered with 100 μL of AEC + substrate-chromogen solution for 10 min, i.e., 3-amino-9-ethylcarbazole containing hydrogen peroxide, stabilizers, enhancers and an anti-microbial agent (Dako, Glostrup, Denmark). After that period the specimen was again rinsed with PBS.
5. The slides were immersed in a bath of aqueous hematoxylin (Mayerr’s hematoxylin), and rinsed gently in a distilled water bath. The slides were dipped 10 times into a bath of ammonia (0.037 mol/L), and then rinsed in a bath of distilled water for four minutes.
6. The specimens were mounted and coverslipped with non-aqueous permanent mounting medium Ultramount.

## Figures and Tables

**Table 1:** Targeted epitope sequences of the human prostate specific antigen (hPSA) molecule, verified in previous studies.

Targeted Sequence	Epitope Scanning	Phage Library	Antibody	Study
**GRHSLFHP**	6mer	10mer	B80	Jette et al. [23]
**LLGRHSLFHPEDTGQV**	15mer		17-1A2	Corey et al. [24]
**RHSLFHP**		15mer	6C8DS	Michel et al. [25]
**RHSLFHPEDTGQ**	15mer, 8mer		H164, 2Cl	Piironen et al. [26]

**Table 2:** Prediction of hPSA epitope **RHSLFHP** was obtained using 10 epitope prediction methods.

Server	Epitope Prediction Method	hPSA Epitope
1	Kyte and Doolittle (Hydrophobicity)	**RHSLFHPED**
2	BcePred (Antigenic propensity)	**NKSVILLGRHSL**
3	BepiPred-2 (Random forest detection)	**FHPED**
4	LBtope (SVM & κ-NN detection)	**LGRHSLFHP**
5	COBEpro (Epitopic propensity)	**PEDTGQV**
7	Sann (Solvent accessibility)	**FHP**
6	RVP-net (Solvent accessibility)	**RHS**––**HP**
8	NetTurnP (β-turn)	**RHSL**
9	LEPS (β-turn)	**RHSLFH**
10	Informational spectrum (ISM—EIIP)	**LFHPE**
	Frequency Consensus (>5)	**111111226666677431111** **NKSVILLGRHSLFHPEDTGQV**

**Table 3:** The antisense peptide sequence AVRDKVG was obtained by joining four paratopes one by one. The paratopes motifs were selected on the basis of the highest number of antibody homologies detected by the Basic Local Alignment Search Tool (BLAST) [45].

hPSA Epitope (aa 53–59)	RHSLFHP	Antibody Motifs (BLAST)
Paratope scan	**AVRD**	8
	**VRDK**	17
	**RDKV**	10
	**DKVG**	73
Antisense paratope (total)	**AVRDKVG**	108

**Table 4:** Quality control of hPSA staining with antisense peptide dilutions, based on NordiQC scoring criteria: 0—Poor; 1—Borderline; 2—Good; 3—Optimal [57].

Patient No.\Dilutions *	1:10	1:50	1:100	1:200	1:500
1	0	1	**2**	0	0
2	0	1	**2**	1	0
3	0	2	**3**	1	0
4	0	1	**2**	1	0
5	0	1	**3**	1	0
6	0	1	**2**	1	0
7	0	1	**2**	1	0
Sum of scores	0	8	**16**	6	0

* The column with the largest scores is marked red.

## Supplementary Materials

Supplementary materials can be found at https://www.mdpi.com/1422-0067/20/9/2090/s1.

## Abbreviations

BLAST	Basic Local Alignment Search Tool
hPSA	Human Prostate Specific Antigen
HRP	Horseradish Peroxidase
IHC	Immunohistochemistry
*K*_d_	Dissociation Constant
mAb	Monoclonal Antibody
MPEIA	Magnetic Particle Enzyme Immunoassay
MST	Microscale Thermophoresis
PDB	Protein Data Bank
PMT	Photomultiplier Tube
RMSD	Root-Mean-Square-Deviation
SEM	Standard Error of the Mean

## Appendix A

**Table A1:** Amino acid pairs of the receptor (blue)–peptide (red) complex, at a distance closer than 4.5 Å, predicted by CABS-dock server for flexible protein–peptide docking [48].

hPSA Region 53–60 (RHSLFHPE)	Antisense Ligand (AVRDKVG)
**L 56**	**V6, G7**
**H 58**	**D4, V6, G7/R3**
**E 60**	**G7**

3 Å ≤ RMSD (distance) ≤ 5.5 Å = medium-quality prediction.

**Table A2:** NordiQC scoring criteria which include staining intensity, signal-to-noise ratio, background staining, aberrant staining pattern, counterstaining and preservation of morphology [57].

Score	Criteria
Optimal (3)	Staining reaction considered perfect or close to perfect.
Good (2)	Staining reaction considered fully acceptable. The protocol may be optimized to ensure the best staining intensity and signal-to-noise ratio.
Borderline (1)	Staining considered insufficient.
Poor (0)	Staining considered very insufficient.
